# Corneal melting after collagen cross-linking for keratoconus: a case report

**DOI:** 10.1186/1752-1947-5-152

**Published:** 2011-04-16

**Authors:** Georgios Labiris, Eleni Kaloghianni, Stavrenia Koukoula, Athanassios Zissimopoulos, Vassilios P Kozobolis

**Affiliations:** 1 Department of Ophthalmology, Democritus University of Thrace, Alexandroupolis, Greece; 2 Eye Institute of Thrace, Alexandroupolis, Greece; 3 Department of Nuclear Medicine, Democritus University of Thrace, Alexandroupolis, Greece

## Abstract

**Introduction:**

Corneal collagen cross-linking is a rather new technique that uses riboflavin and ultraviolet A light for collagen fiber stabilization in keratoconus corneas. Other than reversible side effects, the preliminary results of corneal collagen cross-linking studies suggest that it is a rather safe technique. In this report, we demonstrate a case of corneal melting after corneal collagen cross-linking for keratoconus corneas associated with an acute inflammatory response.

**Case presentation:**

A 23-year-old Caucasian man with keratoconus cornea stage 1 to 2 underwent uneventful corneal collagen cross-linking treatment according to the Dresden protocol. The next day the patient had intense photophobia, watering and redness of the eye, and his visual acuity was limited to counting fingers. Slit lamp biomicroscopy revealed severe corneal haze accompanied by non-specific endothelial precipitates following an acute inflammatory response. Mild inflammation could be detected in the anterior chamber. Moreover, the re-epithelialization process could barely be detected. His corneal state gradually deteriorated, resulting in descemetocele and finally perforation.

**Conclusion:**

In this report, we present a case of a patient with corneal melting after standard corneal collagen cross-linking treatment for keratoconus corneas following an acute inflammatory response. Despite modifying postoperative treatment, elaboration of all apparent associated causes by the treating physicians and undergoing extensive laboratory testing, the patient developed descemetocele, which led to perforation. Our report suggests that further research is necessary regarding the safety of corneal collagen cross-linking in keratoconus corneas.

## Introduction

Keratoconus (KC) is a degenerative non-inflammatory corneal disease. It is usually bilateral and has an incidence of approximately one per 2000 in the general population [1,2]. In the majority of cases, KC starts at puberty and progresses at a variable rate [2]. Eventually, about 20% of KC eyes require penetrating keratoplasty [3]. Corneal collagen cross-linking (CXL) is a rather new therapeutic approach attempting to address KC progression by using riboflavin and ultraviolet A (UVA) radiation. The primary objective of CXL is to stabilize the collagen fiber matrix in KC corneas [4,5]. Beyond reversible side effects that are mainly associated with postoperative infections, preliminary results of CXL studies suggest that it is a rather safe technique [6,7]. Therefore, recent publications indicate that CXL might be used as a therapeutic alternative in a series of other corneal diseases such as infectious keratitis and corneal bullosa [8,9]. Within this context, we present a case report regarding corneal melting after CXL with riboflavin and UVA for KC that eventually required penetrating keratoplasty because of perforation.

## Case presentation

An otherwise healthy 23-year-old Caucasian man was referred to our institute as a potential candidate for CXL. According to his referral documents, the patient had an uneventful medical history, and despite progressive bilateral keratoconus he had no other ophthalmological problems. However, during the past year, he had developed contact lens intolerance.

**Figure 1:**
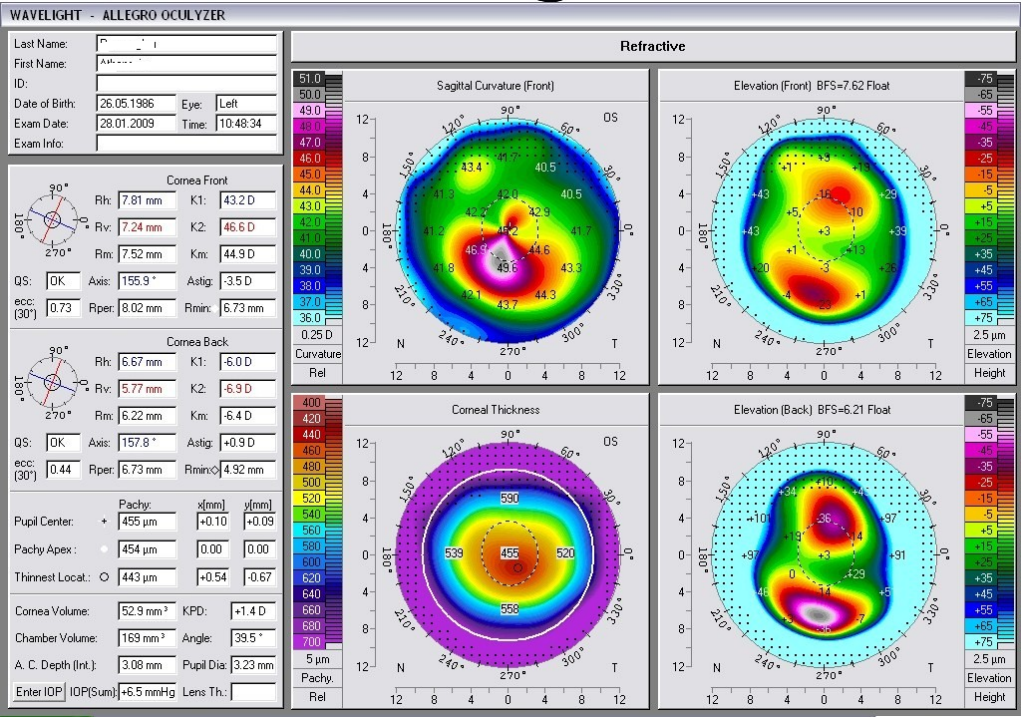
**Preoperative Pentacam Oculyzer image of the patient's left eye**.

At presentation, his uncorrected visual acuities were 0.4 logMar and 0.5 logMar in his right and left eyes, respectively. His best corrected visual acuities (BCVA) were 0.1 logMar (-0.25 spherical (SPH), -2.50 cylindrical (CYL) × 20) in his right eye and 0.3 logMar (-0.50 SPH, -3.00 CYL × 155) in his left eye. Central corneal pachymetry measured with a Scheimpflug camera (Pentacam Oculyzer; Oculus Optikgerate GmbH, Heidelberg, Germany) was 462 μm and 455 μm in his right and left eyes, respectively. The thickness of the thinnest corneal point (TCT) in the left eye was 443 μm (Figure 1), while the keratometric readings derived from the Pentacam test were K1-43.1, K2-46.4 in the right eye and K1-43.2, K2-46.6 in the left eye, respectively. In comparison to the patient's referral documents, within the past year the patient had demonstrated deterioration in his BCVA (former BCVA 0.2 logMar (-0.50 SPH, -2.25 CYL × 155) and in the TCT (former TCT was 449 μm). According to the topographical keratometric data, he was diagnosed with KC stage 1 or 2 and scheduled for CXL therapy.

Standard CXL treatment was performed in the patient's left eye according to the following procedure: Alcaine drops were used for topical anesthesia, followed by application of a sponge saturated with 20% alcohol to the central cornea for 15 seconds. De-epithelialization was performed by means of a hockey knife. The residual corneal thickness after debridement was 407 μm as measured by ultrasound contact pachymetry (Pacline; Optikon 2000 SpA, Rome, Italy). After de-epithelialization, a mixture of 0.1% riboflavin in 20% dextran solution was instilled into the cornea for 30 minutes (two drops every two minutes) until the stroma was completely penetrated and the aqueous humor was stained yellow. Regarding the UVA radiation source, the UV-X system (Peschke Meditrade GmbH, Cham, Switzerland) was employed. An 8.0 mm diameter of the central cornea was irradiated for 30 minutes by UVA light with a wavelength of 370 nm and at surface radiance of 3 mW/cm^2^, which corresponds to a surface dose of 5.4 J/cm^2^. It should be mentioned that the use of riboflavin was continued during irradiation to maintain the necessary concentration. Moreover, balanced salt solution was applied every six minutes to moisten the cornea. When the irradiation was complete, a soft contact lens (Day & Night; CIBA Vision, Duluth, GA, USA) was applied until full re-epithelialization was completed.

The patient was administered the following postoperative medications: (1) gentamicin sulfate and dexamethasone dihydrogenophosphate drops (Dexamytrex Ophtiole; Bausch & Lomb, Berlin, Germany) four times daily and (2) a monodose combination of sodium hyaluronate 0.15% and dexpanthenol 2% (HyloPan; ZwitterPharmaceuticals, Halandri, Greece) every hour.

**Figure 2:**
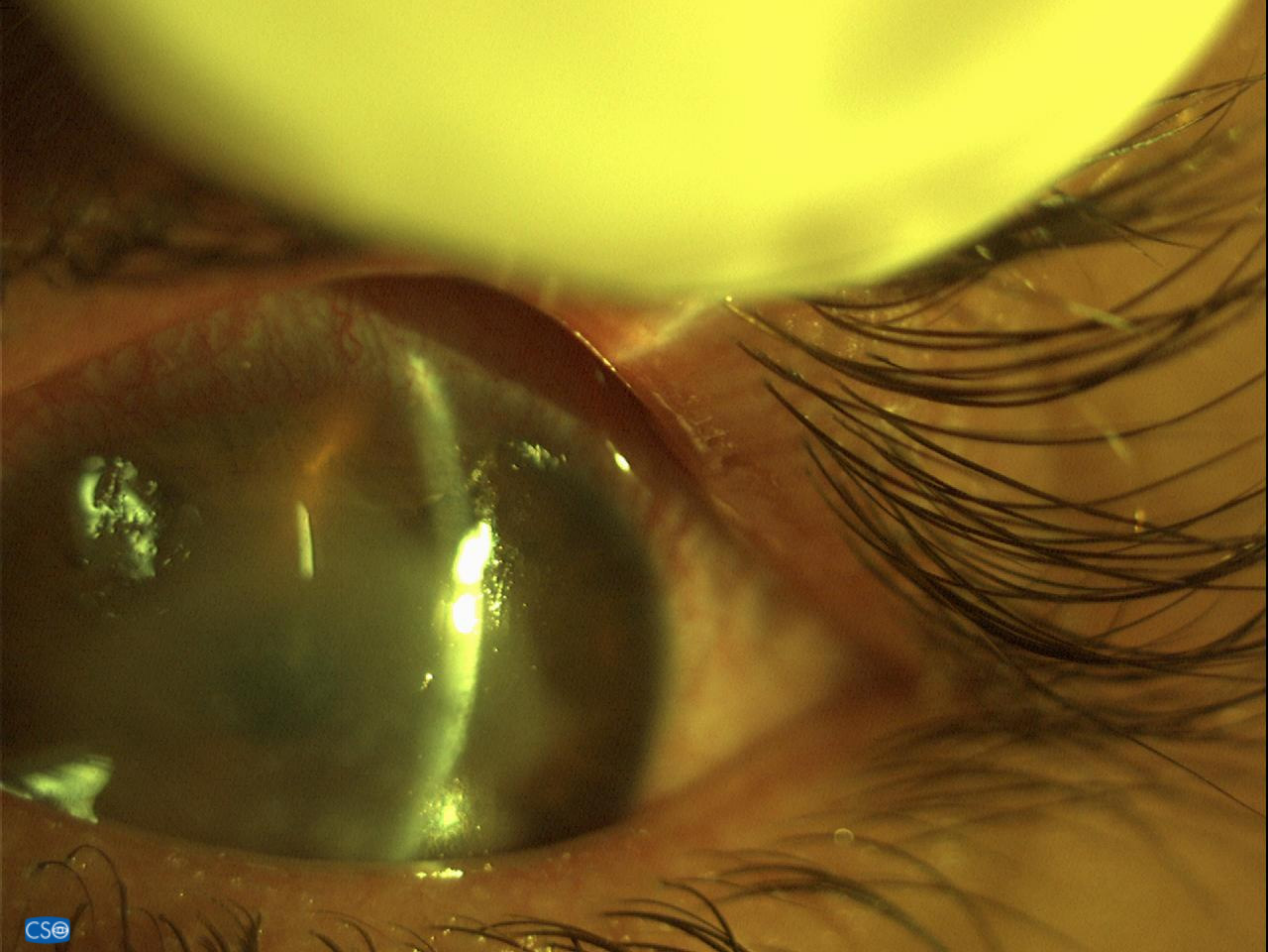
**Slit lamp biomicroscopic image showing severe corneal haze and endothelial precipitates due to the acute inflammatory response**.

Despite an uneventful CXL treatment, during the first postoperative day the patient developed intense photophobia, watering and a non-specific ocular discomfort. Slit lamp biomicroscopy revealed redness, especially at the limbal region, severe corneal haze accompanied by non-specific endothelial precipitates and a few inflammatory cells in the anterior chamber (Tyndall effect +1) (Figure 2). The aforementioned findings resembled an acute inflammatory response to the CXL procedure and/or possibly to the postoperative medication. Moreover, no evidence of re-epithelialization was observed, and the patient's visual acuity was limited to counting fingers.

The patient's postoperative medication was modified to ofloxacin drops four times per day quid (Exocin; Allergan, Castlebar Road, Westport, CoMayo, Ireland), dexamethasone drops every two hours (Maxidex; Alcon Cusi, SA, Spain), frequent use of carboxymethylcellulose 0.5% drops (Optive; Allergan, Irvine, Ca) and oral acyclovir 400 mg four times daily (Zovirax; GlaxoSmithkline, Aranda, Spain). Further to the postoperative regimen change, the patient underwent a complete laboratory examination for autoimmune and infectious diseases, including markers for rheumatoid factor, immune complexes, C-reactive protein, antineutrophilic cytoplasmic antibodies and erythrocyte sedimentation rate, as well as polymerase chain reaction for herpes simplex virus DNA detection, which were all negative or within normal limits. Moreover, repeated cultures from cornea samples and the contact lens were all negative. However, the patient was evaluated for hypersensitivity to riboflavin (vitamin B_2_) and other components of the B vitamin complex as well as a series of common allergens. According to the results presented in Table 1, no evidence of a hypersensitivity reaction could be detected.

**Table 1:** Patient's serum allergen values^a^

RAST test	Immunoglobulin E level, U/mL	
Vitamin B_1_	0.13	(negative)
Vitamin B_2_	0.16	(negative)
Vitamin B_12_	0.18	(negative)
*Dermatophagoides pteronyssinus*	0.09	(negative)
*Dermatophagoides farina*	0.08	(negative)
Erect pellitory-of-the-wall (*Parietaria officinalis*)	0.09	(negative)

^a^RAST test, radioallergosorbent test. (Levels 0-0.35 U/mL negative, 0.35-0.70 U/mL low possibility for allergy, 0.70-3.50 U/mL positive with low immunoglobulin E levels, 3.50-17.50 positive with high immunoglobulin E levels,17.50-52.50 positive with very high immunoglobulin E levels.)

The treatment change resulted in subjective improvement of ocular discomfort and disappearance of the inflammatory cells in the anterior chamber. However, the cornea presented extremely slow re-epithelialization and progressive thinning, which resulted in descemetocele and finally perforation in the second postoperative month. The patient underwent uncomplicated penetrating keratoplasty with an uneventful postoperative period.

## Discussion

Corneal CXL has gained popularity as a temporary block in the progression of keratoconus. Preliminary results published in the literature indicate that when a series of safety precautions are taken, the technique has an excellent safety profile. These prerequisites are (1) de-epithelialization of the cornea to facilitate the absorption of riboflavin, (2) use of riboflavin 0.1% for at least 30 minutes, (3) homogeneous UV irradiation and (4) a minimal central corneal thickness of 400 μm [10]. All of the aforementioned criteria were met in our case.

An extensive literature search retrieved the following cases of CXL melting. Gokhale *et al*. [11] recently presented a case of acute corneal melting after CXL for keratoconus which was attributed to the hazardous impact of diclofenac on stromal keratocytes. Despite the fact that no apparent etiologic relationship between non-steroidal anti-inflammatory drugs (NSAIDs) and corneal melting has been demonstrated in the literature, several investigators have attempted to associate keratolysis with postoperative NSAID therapy [12]. The potential impact of NSAIDs on keratocytes is well known to the authors, thus we did not use NSAIDs as standard postoperative treatment in CXL. Furthermore, Faschinger *et al*. [13] reported a case of bilateral melting after CXL for keratoconus in a patient with Down syndrome; however, the required minimal stromal thickness of 400 μm was not met.

According to our pachymetric data, neither the central corneal thickness nor the thinnest corneal thickness was below 400 μm in our patient. On the other hand, Angunawela *et al*. [14] presented a case of sterile corneal infiltrates and melting after CXL for keratoconus. They attempted to associate their findings with enhanced cell-mediated immunity to staphylococcal antigens deposited at high concentrations in areas with static tear pooling beneath the bandage contact lens. However, the corneal infiltrations were detected five days postoperatively under an intact epithelium. No evidence of non-infective keratitis could be demonstrated in our case. Regarding post-CXL haze, Raiskup *et al*. [15] reported in their retrospective survey that 8.6% of the KC eyes that underwent CXL treatment developed clinically significant permanent stromal haze. However, no associations with increased risk for corneal melting were described.

Concerning potential anaphylaxis with riboflavin, the literature suggests that it is well tolerated even at high doses, and only one documented case of anaphylaxis after oral administration of riboflavin was retrieved [16]. In our patient, no indications of hypersensitivity to riboflavin could be identified.

It is well known that during CXL treatment the keratocytes suffer significant damage because of UV radiation and the generation of oxygen and superoxide radicals [17]. However, the literature suggests that this cell apoptosis is reversible and that the affected area is repopulated within six months [18]. Moreover, because of the shielding effect of riboflavin, the standard CXL procedure seems to cause no damage to the endothelial cells.

## Conclusion

Despite the aforementioned data from other clinical and research settings, the CXL procedure caused non-specific irreversible damage to keratocytes in our patient that cannot be directly attributed to postoperative treatment or to cell-mediated immunity to antigens. Moreover, no evidence of underlying autoimmune disease or local infection could be detected. The exact cause of corneal melting in our case remains unknown to us. An immunohistochemical examination of the affected cornea could provide more data regarding its pathological mechanism. Nevertheless, since all precautions for standard CXL treatment were met in our case, further research is necessary to address all safety issues associated with this procedure.

## Consent

Written informed consent was obtained from the patient for publication of this case report and any accompanying images. A copy of the written consent is available for review by the Editor-in-Chief of this journal.

## Competing interests

The authors declare that they have no competing interests.

## Authors' contributions

GL was involved in the ophthalmic management of the patient and contributed to writing the manuscript. EK performed some of the ophthalmic examinations. SK carried out literature research. AZ performed the general clinical investigation and all hypersensitivity tests. VK was involved in the ophthalmic evaluation of the patient and critically reviewed the paper. All authors read and approved the final manuscript.
